# Spontaneous bowel perforation complicating ventriculoperitoneal shunt: a case report

**DOI:** 10.4076/1757-1626-2-8251

**Published:** 2009-08-07

**Authors:** Theodosios Birbilis, Petros Zezos, Nikolaos Liratzopoulos, Anastasia Oikonomou, Michael Karanikas, Kosmas Kontogianidis, Georgios Kouklakis

**Affiliations:** 1 Department of Neurosurgery, Democritus University of Thrace, University General Hospital, Dragana68100 AlexandroupolisGreece; 2 Gastrointestinal Endoscopy Unit, Democritus University of Thrace, University General Hospital, Dragana68100 AlexandroupolisGreece; 3 1^st^ Department of Surgery, Democritus University of Thrace, University General Hospital, Dragana68100 AlexandroupolisGreece; 4 Radiology Department, Democritus University of Thrace, University General Hospital, Dragana68100 AlexandroupolisGreece

## Abstract

Ventriculoperitoneal shunt placement is an effective treatment of hydrocephalus diverting the cerebrospinal fluid into the peritoneal cavity. Unfortunately, the shunt devices have a high incidence of malfunction mainly due to catheter obstruction or infection and are associated with various complications, 25% of which are abdominal. Spontaneous bowel perforation is a rare potentially fatal complication of ventriculoperitoneal shunt occurring anytime, few weeks to several years, after the placement of the ventriculoperitoneal shunt device. A 54-year-old Greek man with spontaneous perforation of sigmoid colon as a complication of distal ventriculoperitoneal shunt migration was treated successfully by antibiotic prophylaxis and abdominal surgery. Clinicians managing patients with ventriculoperitoneal shunt must be familiar with its possible complications and be aware for early recognition of them.

## Introduction

Ventriculoperitoneal (VP) shunt placement is an effective treatment of hydrocephalus diverting the cerebrospinal fluid (CSF) into the peritoneal cavity. Unfortunately, the shunt devices have a high incidence of malfunction mainly due to catheter obstruction or infection and are associated with various complications, 25% of which are abdominal [1].

Spontaneous bowel perforation is a rare complication of VP shunt, occurring anytime, few weeks to several years, after the placement of the VP shunt device in 0.01% to 0.07% of patients [2]. Colon is the most frequent site of perforation, which may be asymptomatic in almost 50% of cases, but it can result in potentially serious infectious complications, sepsis or even death in ˜15% of cases. Therefore, a high index of suspicion is needed for the early recognition and prompt management of the colonic perforation and its ominous complications [2].

## Case presentation

A 54-year-old Greek man was admitted in the intensive care unit (ICU) with disturbed mental status (Glasgow Coma Score, GCS: 7) due to a severe head injury after a fall accident at work. During his long stay in ICU, almost 6 months, the patient developed secondary post-traumatic hydrocephalus, which was managed with the placement of a programmable Hakim-Medos® ventriculoperitoneal (VP) shunt. After VP shunt surgery the condition of the patient was stabilized and a gradual improvement of his clinical condition and mental status (GCS: 13) was noticed. Consequently, the patient was discharged from ICU and transferred to the Neurosurgical Department for further attention and rehabilitation.

**Figure 1.**
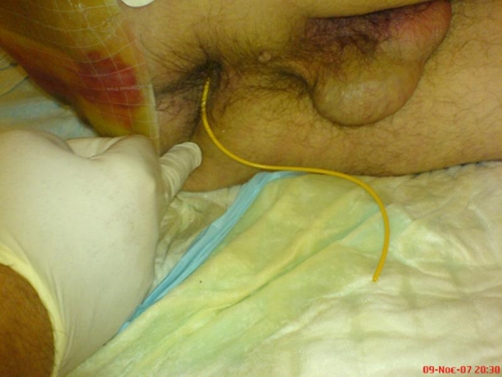
The ventriculoperitoneal shunt catheter is protruding through the patient’s normal-appearing anus.

Seven months after the VP shunt placement, while he was still inpatient, his wife noticed a catheter protruding from his anus after having a bowel movement. The patient was complaining for gradually deteriorating abdominal pain, particularly in the left hypogastrium, during the last week. The patient exhibited no neurologic changes (GCS: 13). He denied nausea, vomiting, melena, hematochezia, urinary urgency, frequency, hematuria, except for pain at the hypogastrium. He was not febrile, there were no signs of meningeal irritation and fundoscopic evaluation was normal with no signs of increased intracranial pressure. The laboratory tests were unremarkable. Rectal examination revealed the VP shunt catheter protruding for a length of 30cm from a normal-appearing anus, with spontaneous retraction in the rectum (Figure 1). There was no gross bleeding.

**Figure 2.**
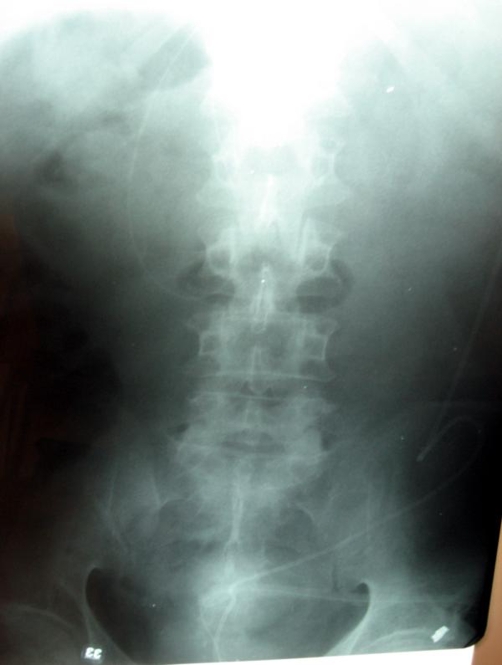
Plain abdominal radiography showing the ventriculoperitoneal shunt catheter within the colonic lumen.

Plain abdominal radiographs showed the distal part of the catheter within the colonic lumen and through the descending and sigmoid colon and the rectum. There was no free air in abdominal cavity (Figure 2).

**Figure 3.**
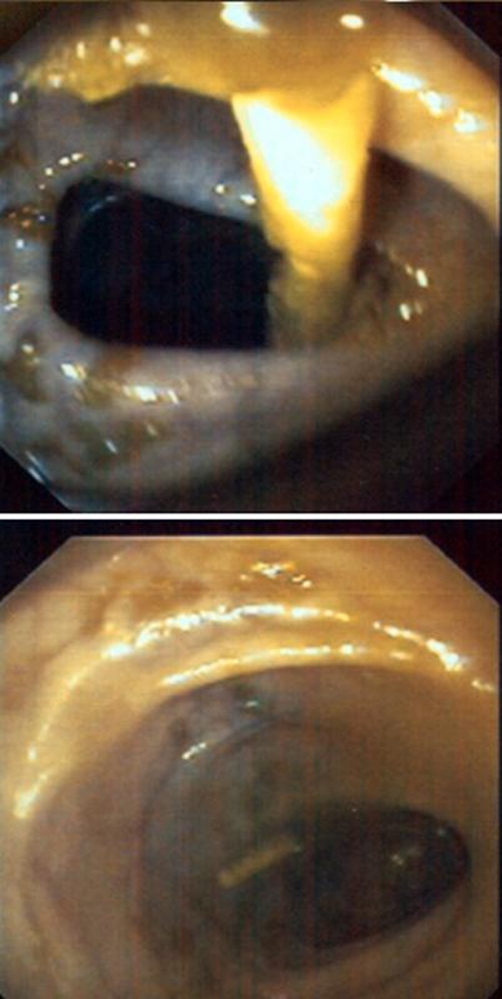
Sigmoidoscopy showed the distal part of the ventriculoperitoneal shunt catheter within the sigmoid colon and the penetration site at the distal descending colon.

Although there were no signs of infectious complications, antibiotic prophylaxis was instituted for bowel perforation with intravenous administration of ciprofloxacin, metronidazole and vancomycin. Since it was difficult for the abdominal plain radiographs to define where the shunt catheter might have penetrated the bowel a flexible sigmoidoscopy was performed for this purpose with cautious air infusion. Sigmoidoscopy showed the catheter located within the sigmoid colon, with the penetration site at the distal descendent colon, approximately 40 cm proximal to the anal verge (Figure 3).

**Figure 4.**
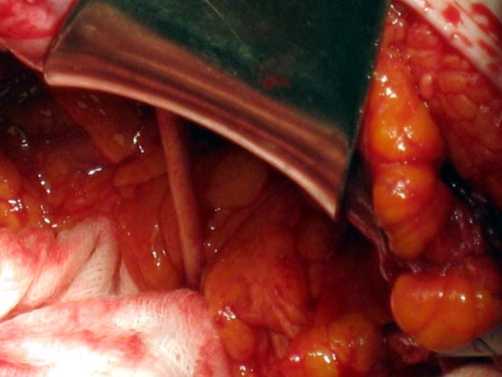
Laparotomy view: the distal part of the ventriculoperitoneal shunt catheter penetrating the sigmoid colon.

We decided to exteriorize the proximal part of peritoneal catheter. The cerebrospinal fluid (CSF) was clear and colorless, while the laboratory examination and the cultures were negative for infection. Laparotomy confirmed that the distal part of the peritoneal catheter had perforated the proximal sigmoid colon with presence of abundant chronic fibrous tissue around the point of perforation (Figure 4). This part of the catheter was then removed through the anus, the fibrous tissue was excised and a primary two-layer closure of the colonic perforation was performed.

The postoperative course was uncomplicated, bowel peristalsis was re-established on day 3, the abdomen was soft, and a normal diet was tolerated by the patient on day 4. Intravenous administration of ciprofloxacin and metronidazole and vancomycin was administered for the next 3 weeks. Six weeks later after repeated negative CSF cultures, the peritoneal part of the AV catheter was placed intra-abdominally, with no complications postoperatively for the following 18 months.

## Discussion

The VP shunt is a standard device used to correct hydrocephalus and its components are a proximal catheter, which is placed into the cerebral ventricle, a valve, and a distal catheter that is placed in the peritoneal cavity.

Ventriculoperitoneal shunt placement is associated with various complications including ventriculitis, meningitis, sepsis, and several rare abdominal complications including intestinal volvulus, pseudocyst, and extrusion through the scrotum, the umbilicus, the vagina, or the gastrointestinal tract. Abdominal complications account for 25% of all complications, can present within a few weeks or several years after the time of surgery, and occur with all types of VP shunt catheters, but are more frequent in the rigid ones [1,2].

Spontaneous bowel perforation is a rare complication of VP shunt surgery, occurring in only 0.01% to 0.07% of patients, most commonly in children, although Vinchon *et al.* reported a higher rate of bowel perforation in their series; almost 1% (19 perforations in 1956 peritoneal shunts) [2,3]. The bowel perforation may have an insidious course since less than 25% of the patients’ exhibit signs of peritonitis, but due to its high mortality rate, it is important for the clinicians to recognize its occurrence when they manage patients with VP shunts. As a rule, any patient with VP shunt who presents with ventriculitis or meningitis due to an enteric organism should be assessed for bowel perforation [2].

Perforation can occur in any segment of the GI tract but the colon is the most commonly reported site. In most of the cases the patient was either asymptomatic or presented with a catheter protruding through the anus or the mouth [2,4]. Symptoms related to bowel perforation included abdominal pain, vomiting, and diarrhea.

The overall mortality rate after perforation is relatively high, approaching 15-18%, and it is further increased when infection is present. Mortality is about 22% with central nervous system (CNS) infection and 33% with intra-abdominal infection, which can result in meningitis, encephalitis, or brain abscesses [4]. CSF cultures are positive in nearly 50% of cases, with *Escherichia coli* being the most common organism.

The etiology of the bowel perforation is unclear, and several mechanisms have been suggested. The formation of a local inflammatory reaction or fibrosis around the distal catheter is thought to have an anchoring effect on the tube resulting in pressure on an area of the bowel which finally causes perforation of the wall. The type of catheter or the length of the abdominal part of the catheter may also be implicated in the bowel perforation and finally silicon allergy may result in a foreign body-like reaction [2].

The diagnosis of the bowel perforation is easy and straightforward when the catheter protrudes through the anus or mouth, although in some cases, contrast injection into the distal part of the catheter may be necessary to confirm it. Abdominal radiology, including plain X-ray and CT scan, are useful diagnostic modalities, while endoscopy may be useful to show the site of catheter penetration through the colonic wall, which can be irregular, friable, or ulcerated [2-4].

The treatment of a VP shunt perforating the bowel is a medical emergency. The perforating part of the catheter must be removed and an external drainage of the proximal part is needed together with antibiotic prophylaxis. In general, there are three methods by which the catheter can be removed: by pulling it through the anus, by endoscopic removal, or by surgical removal. Nevertheless, the management of the bowel perforation must be individualized. The shunt is externalized at its upper end and, once the CSF cultures are negative, a new peritoneal shunt catheter can be placed intra-abdominally few weeks later. If there is no accompanying peritonitis or abdominal abscess, then percutaneous or endoscopic removal of the abdominal shunt catheter can be performed without surgery [2,5,6]. The fibrous tissue surrounding the perforation does not permit the spillage of bowel contents into the peritoneal cavity [6]. Laparotomy must be performed in cases of intra-abdominal infection (peritonitis or abscess) or when the fistulous tract does not close spontaneously after percutaneous or endoscopic removal [2,4,6].

In our patient, the diagnosis of the bowel perforation was easy since the distal part of the ventriculoperitoneal shunt catheter was protruding through the anus. Laboratory and imaging examinations confirmed that the bowel perforation was not complicated with abdominal or intracranial infection, and the prompt institution of antibiotic treatment together with the surgical removal of the catheter both contributed to the favorable outcome.

## Conclusion

It is evident that when the bowel perforation is detected and corrected at an early and asymptomatic stage, the prognosis is excellent. On the other hand this complication can turn to be fatal when infectious complications develop.

Clinicians managing patients with VP shunt must be familiar with its possible complications and be aware for early recognition of the bowel perforation in such patients, especially in asymptomatic cases without protrusion of the catheter through the anus.

## Abbreviations

CNS: central nervous system
CSF: cerebrospinal fluid
CT: computer tomography
GCS: Glasgow Coma Score
ICU: intensive care unit
VP: ventriculo-peritoneal
